# Risk factors and a nomogram for predicting severe acute kidney injury in pediatric sepsis: a retrospective study

**DOI:** 10.3389/fped.2026.1822958

**Published:** 2026-07-20

**Authors:** Yiru Xiang, Ping Zang, Runfang Chen, Haipeng Yan, Xun Li, Jun Qiu, Zhenghui Xiao, Xiulan Lu

**Affiliations:** 1 The School of Pediatrics, Hengyang Medical School, University of South China (Hunan Children's Hospital), Hengyang, China; 2 Department of Pediatric Intensive Care Unit (PICU), The Affiliated Children's Hospital of Xiangya School of Medicine, Central South University (Hunan Children's Hospital), Changsha, China; 3 Pediatrics Research Institute of Hunan Province, The Affiliated Children's Hospital of Xiangya School of Medicine, Central South University (Hunan Children's Hospital), Changsha, China

**Keywords:** acute kidney injury, children, nomogram, risk factor, sepsis

## Abstract

**Objective:**

To develop and validate a nomogram for predicting the risk of severe acute kidney injury (AKI) in children with sepsis.

**Methods:**

A total of 987 children with sepsis admitted to the pediatric intensive care unit (PICU) of Hunan Children's Hospital between July 2018 and January 2021 were enrolled. Patients were stratified into a severe AKI group (*n* = 228) and a no clinically significant AKI group (*n* = 759) according to the severity of AKI during hospitalization. Based on independent risk factors identified by multivariate logistic regression, a predictive nomogram was constructed. Model performance was evaluated using receiver operating characteristic (ROC) curves, calibration curves, and decision curve analysis (DCA).

**Results:**

Severe AKI occurred in 228 patients (23.1%). The mortality rate in the severe AKI group was 2.57-fold higher than that in the no clinically significant AKI group (31.1% vs. 12.5%, *P* < 0.05). Independent predictors of severe AKI included elevated phosphate (P^5+^; per 1 mmol/L increase: *OR* = 2.789, *95% CI* = 1.693–4.592, *P* < 0.001), decreased albumin (ALB; per 1 g/L increase: *OR* = 0.930, *95% CI* = 0.879–0.984, *P* = 0.012), elevated uric acid (UA; per 1 μmol/L increase: *OR* = 1.004, *95% CI* = 1.003–1.005, *P* < 0.001), and reduced antithrombin III (AT3; per 1% increase: *OR* = 0.990, *95% CI* = 0.980–0.999, *P* = 0.048). The logistic regression model showed moderate discrimination with an area under the ROC curve (AUC) of 0.782 (*95% CI* = 0.744–0.819). The corresponding nomogram achieved an AUC of 0.761 (*95% CI* = 0.724–0.797). At their optimal cutoff values, the model and nomogram yielded sensitivities of 60.10% and 62.70%, specificities of 85.40% and 80.80%, and Youden indices of 0.455 and 0.435, respectively.

**Conclusion:**

Severe AKI is strongly associated with increased mortality in pediatric sepsis. The nomogram incorporating P^5+^, ALB, UA, and AT3 shows moderate discrimination, acceptable calibration, and potential clinical utility for predicting severe AKI in children with sepsis.

## 1 Introduction

Sepsis is a life-threatening organ dysfunction syndrome caused by a dysregulated host response to infection, and remains one of the most common conditions in the intensive care unit (ICU) (1). In the Pediatric Intensive Care Unit (PICU), approximately 26.9% of children with sepsis may develop acute kidney injury (AKI), with rapid disease progression and a mortality rate as high as 3.4% (2, 3).

Recent studies have shown that among children with sepsis in the PICU, those who develop severe AKI (KDIGO stages 2 and 3) have a mortality rate or probability of disability twice that of children with mild AKI (stage 1) or no AKI (4). Moreover, the risk of readmission within one year after discharge increases by 40%, and the risk within five years increases by 60% to 80% (5), imposing a substantial burden on affected children and their families. In the pathogenesis of sepsis-associated AKI, the vicious cycle formed by aberrant activation of the coagulation system and the inflammatory response constitutes a critical pathway (5). Sepsis-induced coagulopathy is characterized by systemic microvascular thrombosis, which directly contributes to renal ischemia through obstruction of renal blood flow and exacerbation of endothelial injury (6). Clinical observations further confirm that levels of markers reflecting coagulation disturbances are closely associated with disease severity. For instance, Li et al. (7) demonstrated that elevated D-dimer and FDP levels are not only sensitive indicators of coagulation activation but also independent prognostic factors in sepsis patients. Beyond coagulation abnormalities, electrolyte disturbances also pose significant threats (8); previous studies have confirmed that abnormal levels of potassium, chloride, calcium, and phosphate are significantly associated with increased mortality in sepsis patients (9). In addition, comprehensive clinical indicators including the fibrinogen-to-albumin ratio (FAR), serum creatinine, and SOFA score have been established as independent predictors of 28-day mortality in patients with sepsis-associated AKI (10, 11).

Current research on AKI in sepsis predominantly focuses on adult populations, or simply compares AKI vs. non-AKI without distinguishing severity, despite the limited prognostic impact of mild AKI. Studies specifically addressing severe AKI in children remain scarce. Therefore, using data from the only pediatric specialty hospital in Hunan Province (a regional medical center), this study aimed to identify risk factors for severe AKI in children with sepsis in the PICU and to establish a reliable predictive model to inform clinical practice.

## 2 Materials and methods

### 2.1 Study subjects

The study was approved by the Medical Ethics Committee of Hunan Children's Hospital (Approval No. HCHLL-2021-123). Children with sepsis who were admitted to the pediatric intensive care unit (PICU) of Hunan Children's Hospital from July 2018 to January 2021 were recruited into our study. Inclusion criteria were: (1) Age > 28 days and <18 years. (2) Length of stay in the PICU > 24 h. (3) Meeting the diagnostic criteria for sepsis (1). Exclusion criteria were: (1) AKI occurring before admission to the PICU. (2) History of kidney transplantation, chronic kidney disease, or long-term dialysis. (3) Death within 24 h or withdrawal of treatment due to family reasons. (4) Missing data.

### 2.2 Diagnostic criteria and classification

The diagnosis of pediatric sepsis followed the definitions proposed by the 2005 International Pediatric Sepsis Consensus Conference (IPSCC), which include the presence of systemic inflammatory response syndrome (SIRS) with suspected or confirmed infection and organ dysfunction (12).

For children, the baseline creatinine levels are defined as follows: for children under 1 year of age, the creatinine predictive values reported by Boer et al. (13). are used; for children over 1 year of age, the following formula is applied (6):

Average creatinine = −2.37330 − 12.91367 × loge(age) + 23.93581 × (age)^(1/2) (μmol/L).

According to the KDIGO criteria (14), acute kidney injury (AKI) is defined as follows, with the highest serum creatinine (SCr) value recorded after admission used to determine whether the criteria were met in this study: (1) An increase in SCr of ≥0.3 mg/dL (≥26.5 μmol/L) within 48 h; (2) Known or presumed kidney injury occurring within 7 days, with SCr rising to ≥1.5 times the baseline value; (3) Urine output <0.5 mL/(kg·h) for a duration of ≥6 h.

The classification standards for AKI are as follows (15): AKI 1: SCr levels 1.5–1.9 times the baseline, or an increase of ≥0.3 mg/dL (26.5 μmol/L); AKI 2: SCr levels 2.0–2.9 times the baseline; AKI 3: SCr levels greater than 3 times the baseline, or SCr ≥4 mg/dL (353.6 μmol/L), or the initiation of renal replacement therapy.

Patients who developed severe AKI during hospitalization for sepsis were assigned to the AKI 2–3 Group, while the remaining patients were classified into the No-AKI/AKI 1 Group.

Patients who developed AKI stage 2–3 during sepsis hospitalization were assigned to the “Severe AKI Group”, whereas those with no AKI or AKI stage 1 were assigned to the “No clinically significant AKI Group”.

### 2.3 Data collection

Clinical data were obtained from the electronic medical record system for all pediatric patients. The collected information comprised general demographics (age, sex, survival status, and length of hospital stay); disease severity scores [Pediatric Critical Illness Score (PCIS) and Pediatric Sequential Organ Failure Assessment (pSOFA)]; clinical comorbidities and interventions (including Septic shock, use of dopamine or epinephrine, mechanical ventilation, plasma exchange, hemoperfusion, continuous plasma purification, and hemofilter support); and laboratory parameters measured within 24 h of PICU admission. These laboratory parameters included a complete blood count (encompassing red blood cell count [RBC], white blood cell count [WBC] with differentials [neutrophils (NE), monocytes (MO), lymphocytes (LY), eosinophils (EO), and basophils (BA)], platelet count [PLT], and hemoglobin [HB]), inflammatory markers [C-reactive protein [CRP] and procalcitonin [PCT]], electrolytes (sodium [Na⁺], chloride [Cl⁻], calcium [Ca^2^⁺], potassium [K⁺], magnesium [Mg^2^⁺], and phosphate [P^5+^]), liver function tests (total protein [TP], albumin [ALB], total bilirubin [TBIL], direct bilirubin [DBIL], indirect bilirubin [IBIL], aspartate aminotransferase [AST], alanine aminotransferase [ALT], total bile acids [TBAC], AST/ALT ratio, albumin/globulin ratio [A/G], and globulin ), renal function parameters (serum creatinine [SCr], uric acid [UA], blood urea nitrogen [BUN], and the BUN/creatinine ratio [BUN/CREA]), myocardial injury markers (myoglobin [MB], creatine kinase [CK], creatine kinase-MB isoenzyme [CK-MB], and lactate dehydrogenase [LDH]), and coagulation profiles (activated partial thromboplastin time [APTT], prothrombin time [PT], fibrinogen [FIB], thrombin time [TT], international normalized ratio [INR], antithrombin III [AT3], fibrin degradation products [FDP], and D-dimer [D-D]). For renal function assessment, both the initial serum creatinine value within 24 h of PICU admission and the peak serum creatinine level during hospitalization were recorded.

### 2.4 Statistical methods

Data analysis was performed using the statistical software SPSS 26.0 (IBM) and R 4.2.2 software. A total of 58 variables were included in this study, of which 28 had missing data, all with missing rates below 10% (Supplementary Table S1). For variables with missing data rates below 10%, we employed the randomForest package in R for imputation on the entire dataset (without training-test split). The imputed and cleaned dataset was then imported into SPSS for subsequent statistical tests. Categorical data were analyzed using the chi-square test (*χ*^2^-test) and presented as counts (percentages). For continuous data that followed a normal distribution with equal variances, the *t*-test was employed, and results were expressed as mean ± standard deviation ($\bar{x}\pm s$). For continuous data that did not follow a normal distribution or had unequal variances, the median (M) and interquartile range (IQR) were reported.

Multivariate logistic regression analysis was conducted to identify risk factors for the occurrence of severe AKI in pediatric patients with sepsis during hospitalization. A predictive model was established based on the independent risk factors. The model's discriminative ability, stability, calibration performance, and clinical utility were comprehensively evaluated using receiver operating characteristic (ROC) curve analysis, bootstrap resampling, calibration curves, and decision curve analysis (DCA). After reassigning values to the independent risk factors, the rms package was utilized to construct a nomogram prediction model. The predictive accuracy and reliability of the nomogram were validated using the same evaluation framework (ROC analysis, calibration curves, bootstrap resampling, and DCA). A *p*-value of <0.05 was considered statistically significant.

## 3 Result

### 3.1 General information

**Figure 1:**
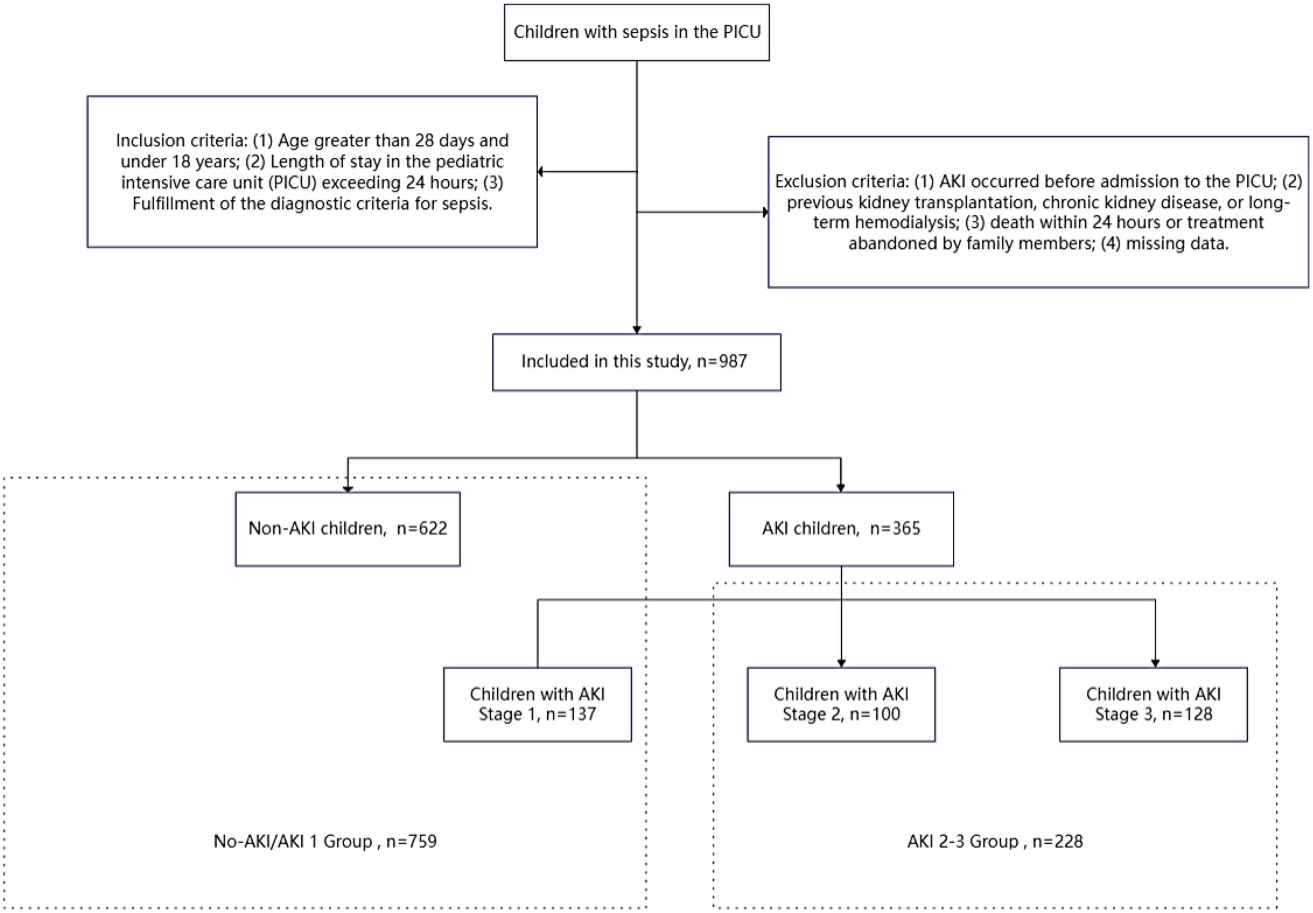
Study population flowchart. AKI, acute kidney injury.

A total of 987 pediatric sepsis patients were enrolled. Among them, 228 (23.1%) developed severe AKI, while 759 (76.9%) had no or mild AKI (No clinically significant AKI Group) (Figure 1). The median age of the patients was 1.20 years (interquartile range: 0.50, 3.92). The overall mortality rate was 16.8% (166/987). The mortality rate in the Severe AKI Group was 31.1%, which was 2.57 times higher than that in the No clinically significant AKI Group, demonstrating a statistically significant difference (*P* < 0.05).

Furthermore, patients in the Severe AKI Group had significantly higher pSOFA scores and rates of hemofilter support compared to the No clinically significant AKI Group (*P* < 0.05). In contrast, the PCIS scores, dopamine usage, mechanical ventilation rate, and continuous plasma purification rate were significantly lower in the Severe AKI Group (*P* < 0.05). Although the plasma exchange rate was numerically similar between the two groups, the difference reached statistical significance (*P* < 0.05). No statistically significant differences were observed between the groups in terms of gender distribution, hospital stay length, incidence of septic shock, epinephrine usage, or hemoperfusion rate (*P* > 0.05). (Table 1 and Supplementary Table S2).

**Table 1:** Comparison of general information between severe AKI group and No clinically significant AKI group.

General Information	No clinically significant AKI	Severe AKI	*P* Value
Age Group			0.256
Infancy (28 days–1 year)	471 (75.8%)	150 (24.2%)	
Childhood (2–10 years)	253 (79.9%)	64 (20.1%)	
Adolescence (11–18 years)	35 (71.4%)	14 (28.6%)	
Gender(n)			0.794
Female	289 (76.5%)	89 (23.5%)	
Male	470 (77.2%)	139 (22.8%)	
Prognosis(*n*)			<0.001
Survived	664 (87.5%)	157 (68.9%)	
Died	95 (12.5%)	71 (31.1%)	
Length of Hospital Stay(day)	15.00 (10.00, 26.00)	19.00 (10.00, 32.50)	0.053
PCIS (score)	90.81 ± 5.78	87.12 ± 7.31	<0.001
pSOFA(score)	4.94 ± 3.36	7.63 ± 4.43	<0.001
Septic Shock(n)			0.197
no	688 (77.5%)	200 (22.5%)	
yes	71 (71.7%)	28(28.3%)	

PCIS, pediatric critical illness score; pSOFA, pediatric sequential organ failure assessment.

### 3.2 The comparison of laboratory indicators between two groups

Compared with the No clinically significant AKI Group, the Severe AKI Group showed significantly lower levels of NE, BA, PLT, Ca^2^⁺, TP, ALB, FIB, and AT3 (*P* < 0.05), while demonstrating significantly elevated levels of PCT, K⁺, Mg^2^⁺, P^5+^, AST, ALT, AST/ALT, SCr, UA, BUN, MB, CK, CK-MB, LDH, APTT, PT, TT, INR, FDP, and D-D (*P* < 0.05). However, no statistically significant intergroup differences were observed in RBC, WBC, MO, LY, EO, HB, CRP, Na⁺, Cl⁻, TBIL, DBIL, IBIL, TBAC, A/G, GLO, or BUN/CREA (*P* > 0.05) (Table 2 and Supplementary Table S3).

**Table 2:** Comparison of laboratory indicators between severe AKI group and No clinically significant AKI group.

**Variable**	**No clinically significant AKI**	**Severe AKI**	***P* Value**
CRP (mg/L)	55.89 ± 66.11	64.49 ± 66.46	0.085
PCT (ng/mL)	1.36 (0.34, 6.68)	7.88 (1.26, 31.70)	＜0.001
Na^+^(mmol/L)	134.00 (132.00, 136.00)	134.00 (129.00, 136.00)	0.122
Cl^−^(mmol/L)	101.70 (99.04, 103.50)	100.90 (97.52, 104.10)	0.069
Ca^2+^(mmol/L)	2.16 (2.03, 2.28)	2.04 (1.86, 2.26)	<0.001
K^+^(mmol/L)	4.15 (3.77, 4.60)	4.30 (3.86, 4.92)	0.001
Mg^2+^(mmol/L)	0.85 (0.77, 0.94)	0.88 (0.77, 0.992)	0.006
P^5+^(mmol/L)	1.17 (0.97, 1.42)	1.41 (1.13, 1.87)	<0.001
TP(g/L)	56.70 (50.53, 62.38)	54.25 (47.63, 59.90)	＜0.001
ALB(g/L)	34.20 (30.20, 37.60)	32.10 (29.20, 36.45)	0.024
SCr (μmol/L)	25.00 (20.00, 32.00)	53.50 (30.00, 90.93)	<0.001
UA(μmol/L)	200.00 (136.00, 277.00)	383.00 (199.00, 654.25)	<0.001
BUN (mmol/L)	3.45 (2.50, 4.78)	8.01 (3.87, 14.37)	<0.001
BUN/CREA	0.15 ± 0.08	0.15 ± 0.08	0.749
MB (ng/mL)	45.30 (21.20, 115.10)	110.40 (40.08, 555.92)	<0.001
CK(U/L)	81.00 (40.00, 180.00)	170.00 (53.75, 750.75)	<0.001
CK-MB(U/L)	17.20 (10.30, 26.10)	29.35 (12.83, 80.85)	<0.001
LDH(u/L)	430.00 (299.50, 748.50)	681.00 (364.75, 1,329.50)	<0.001
APTT(s)	43.70 (38.20, 50.90)	46.40 (39.20, 56.50)	<0.001
PT(s)	14.30 (13.20, 15.70)	15.90 (14.03, 19.70)	<0.001
FIB (mg/dL)	362.00 (261.00, 497.50)	318.50 (214.75, 469.25)	0.006
TT(s)	16.30 (15.00, 18.40)	17.65 (15.60, 21.10)	<0.001
INR	1.12 (1.01, 1.26)	1.28 (1.10, 1.68)	<0.001
AT3(%)	91.00 (71.00, 108.00)	75.00 (60.00, 95.00)	0.001
FDP(μg/mL)	9.81 (6.02, 19.31)	16.88 (8.08, 40.62)	<0.001
D-D(μg/mL)	1.14(0.51, 2.49)	1.79(0.87, 7.15)	<0.001

CRP, C-reactive protein; PCT, procalcitonin; Na⁺, sodium; Cl⁻, chloride; Ca^2^⁺, calcium; K⁺, potassium; Mg^2^⁺, magnesium; P^5^⁺, phosphate; TP, total protein; ALB, albumin; SCr, serum creatinine; UA, uric acid; BUN, blood urea nitrogen; BUN/CREA, BUN to creatinine ratio; MB: myoglobin; CK, creatine kinase; CK-MB, creatine kinase isoenzyme MB; LDH, lactate dehydrogenase; APTT, activated partial thromboplastin time; PT, prothrombin time; FIB, fibrinogen; TT, thrombin time; INR, international normalized ratio; AT3, antithrombin III; FDP, fibrin degradation products; D-D: D-dimer.

### 3.3 Analysis of risk factors for severe AKI in children with sepsis

Univariate analysis of laboratory parameters identified the following factors as significantly associated with severe AKI (*P* < 0.05): NE, BA, PLT, PCT, Ca^2^⁺, K⁺, Mg^2^⁺, P^5+^, TP, ALB, AST, ALT, AST/ALT ratio, SCr, UA, BUN, MB, CK, CK-MB, LDH, APTT, PT, FIB, TT, INR, AT3, FDP, D-Dimer, PCIS, pSOFA. Subsequent multivariate regression analysis revealed that each 1 mmol/L increase in P^5+^ (*OR* = 2.789, *95% CI*: 1.693–4.592, *P* < 0.001) and each 1 μmol/L increase in UA (*OR* = 1.004, *95% CI*: 1.003–1.005, *P* < 0.001) were independent risk factors for severe AKI. In contrast, each 1 g/L increase in ALB (*OR* = 0.930, *95% CI*: 0.879–0.984, *P* = 0.012) and each 1% increase in AT3 (*OR* = 0.990, *95% CI*: 0.980–0.999, *P* = 0.048) were independent protective factors, indicating that higher levels of albumin and antithrombin III were associated with a reduced risk of severe AKI (Table 3).

**Table 3:** Logistic analysis of risk factors for severe AKI children with sepsis.

Variable	Univariate Logistic Regression Analysis			Multivariate Logistic Regression Analysis		
	*OR*	*95%CI*	*P* Value	*OR*	*95%CI*	*P* Value
NE (10^-3)	1	(1.000,1.000)	0.022			
BA (10^-3)	1.047	(1.007,1.089)	0.022			
PLT (10^9/L)	0.999	(0.998, 1.000)	0.008			
PCT (ng/mL)	1.033	(1.025, 1.042)	<0.001			
Ca^2+^(mmol/L)	0.084	(0.042, 0.168)	<0.001			
K^+^(mmol/L)	1.611	(1.327, 1.956)	<0.001			
Mg^2+^(mmol/L)	61.410	(8.030, 469.614)	<0.001			
P^5+^(mmol/L)	4.710	(3.349, 6.625)	<0.001	2.789	(1.693, 4.592)	<0.001
TP(g/L)	0.947	(0.917, 0.978)	0.001			
ALB(g/L)	0.946	(0.921, 0.971)	<0.001	0.930	(0.879, 0.984)	0.012
AST(U/L)	1.001	(1.000, 1.001)	<0.001			
ALT(U/L)	1.000	(1.000, 1.001)	0.001			
AST/ALT	1.002	(0.999, 1.004)	0.271			
UA(μmol/L)	1.005	(1.004, 1.006)	<0.001	1.004	(1.003, 1.005)	<0.001
BUN(mmol/L)	1.722	(1.509, 1.966)	<0.001			
MB(ng/mL)	1.003	(1.002, 1.003)	<0.001			
CK(U/L)	1.000	(1.000, 1.000)	0.003			
CK-MB(U/L)	1.002	(1.001, 1.003)	0.002			
LDH(u/L)	1.001	(1.001, 1.001)	<0.001			
APTT(s)	1.030	(1.012, 1.049)	0.001			
PT(s)	1.133	(1.093, 1.174)	<0.001			
FIB (mg/dL)	0.999	(0.998, 1.000)	0.029			
TT(s)	1.033	(1.016, 1.05)	<0.001			
INR	2.839	(2.059, 3.916)	<0.001			
AT3(%)	0.981	(0.970, 0.992)	0.001	0.990	(0.980, 0.999)	0.048
FDP(μg/mL)	1.006	(1.003, 1.009)	<0.001			
D-D(μg/mL)	1.036	(1.007, 1.067)	0.014			
PCIS (score)	0.914	(0.893, 0.936)	<0.001			
pSOFA (score)	1.198	(1.151, 1.248)	<0.001			

NE, eosinophil count; BA, basophils; PLT, platelet count; PCT, procalcitonin; Ca^2^⁺, calcium; K⁺, potassium; Mg^2^⁺, magnesium; P^5+^, phosphate; TP, total protein; ALB, albumin; AST, aspartate aminotransferase; ALT, alanine aminotransferase; AST/ALT, aspartate aminotransferase to alanine aminotransferase ratio; UA, uric acid; BUN, blood urea nitrogen; MB, myoglobin; CK, creatine kinase; CK-MB, creatine kinase-MB isoenzyme; LDH, lactate dehydrogenase; APTT, activated partial thromboplastin time; PT, prothrombin time; FIB, fibrinogen; TT, thrombin time; INR, international normalized ratio; AT3, antithrombin III, FDP, fibrin degradation products; D-D, D-dimer; PCIS, Pediatric Critical Illness Score; pSOFA, pediatric Sequential Organ Failure Assessment.

### 3.4 Prediction model and ROC curve analysis

The regression model includes four variables: P^5+^ (X₁), ALB (X_2_), UA (X_3_), AT3 (X_4_). The No clinically significant AKI Group is designated as 0, while the Severe AKI Group is designated as 1. The resulting prediction model is given by the equation:Logit(P) = −0.9121 + 1.056X₁ − 0.0716X_2_ + 0.0046X_3_ − 0.0077X_4_.

**Figure 2:**
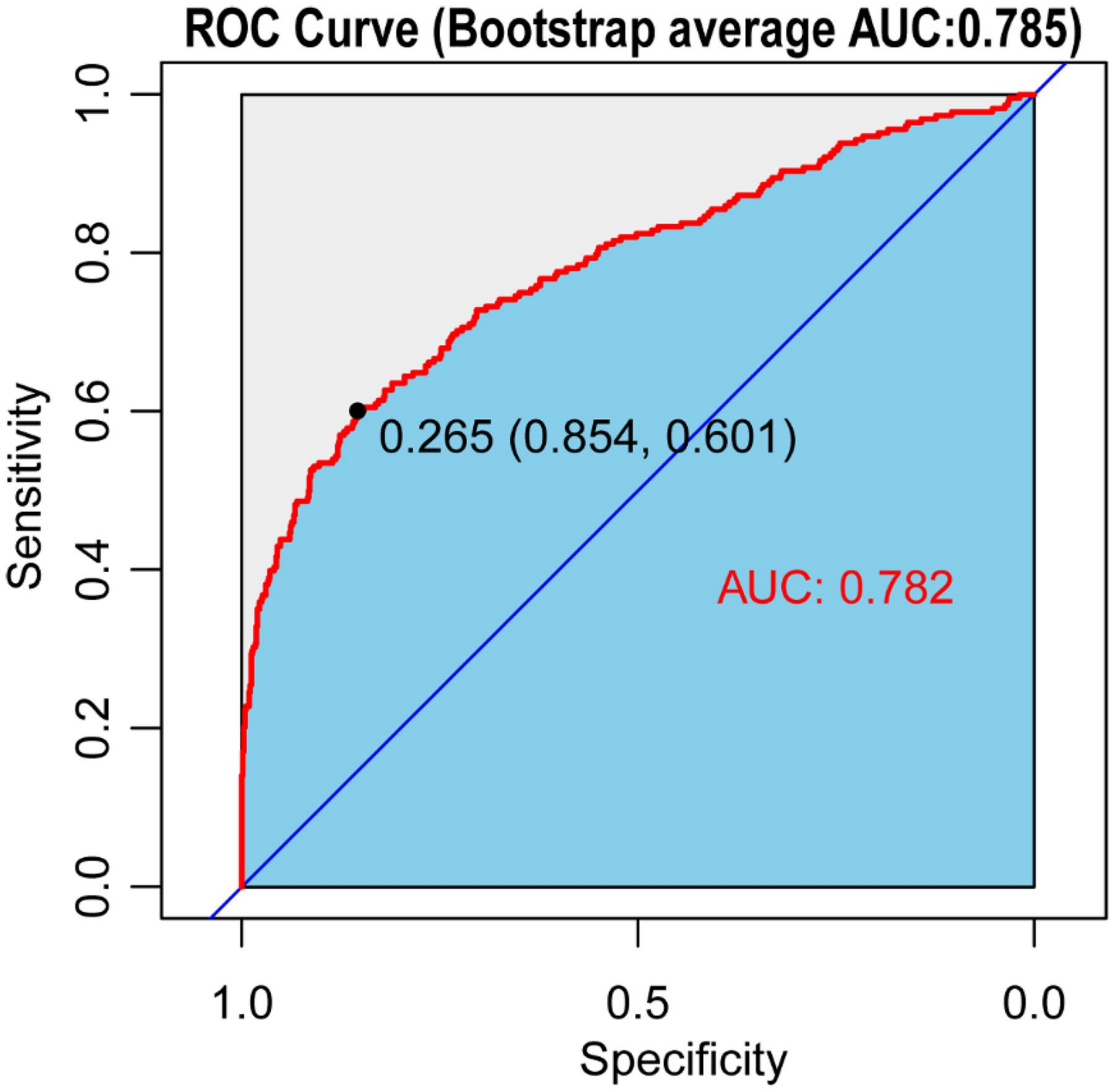
ROC curve for the combined prediction of severe AKI pediatric patients with sepsis using P^5+^, ALB, UA, AT3.

**Figure 3:**
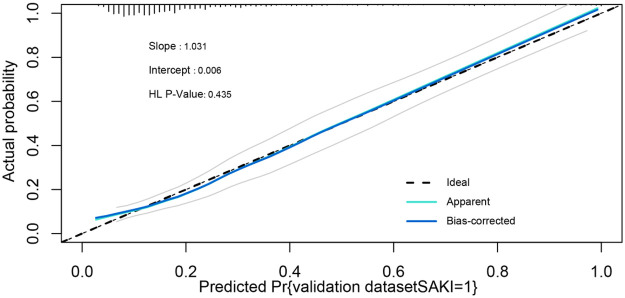
Calibration curve for the combined prediction of severe AKI pediatric patients with sepsis using P^5+^, ALB, UA, AT3. SAKI, severe acute kidney injury.

**Figure 4:**
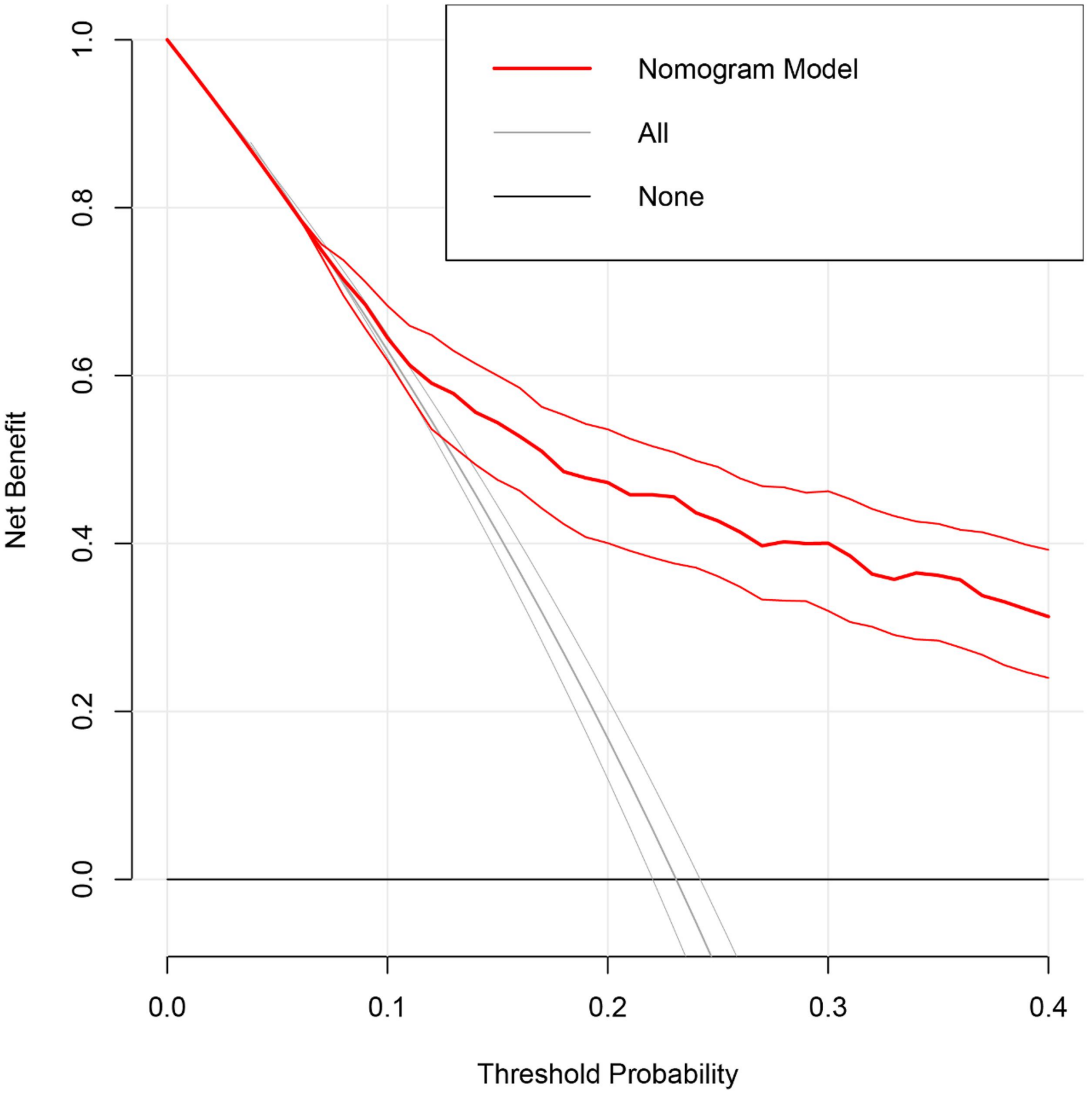
The DCA curve of the prediction model.

The discriminative ability of the model was assessed using receiver operating characteristic (ROC) curve analysis, which yielded an area under the curve (AUC) of 0.782 (*95% CI*: 0.744–0.819). Internal validation through 1,000 bootstrap resamples demonstrated a mean AUC of 0.785 (*95% CI*: 0.748–0.821). At the optimal cutoff value of 0.265, the model achieved a Youden's index of 0.455, with sensitivity of 60.10%, specificity of 85.40%, positive predictive value of 55.20%, negative predictive value of 87.70%, positive likelihood ratio of 4.11, and negative likelihood ratio of 0.467 (Figure 2). The calibration curve showed that the bias-corrected curve closely aligned with the ideal diagonal line, with a calibration slope of 1.031 and intercept of 0.006, and the Hosmer-Lemeshow test yielded a *P* value of 0.435, indicating good agreement between predicted probabilities and observed event rates (Figure 3). Decision curve analysis demonstrated that within the threshold probability range of 0–0.4, the nomogram provided higher net benefit than both the “treat-all” and “treat-none” strategies, suggesting favorable clinical utility (Figure 4).

### 3.5 Binary assignment of predictive variables and presentation of the nomogram

**Figure 5:**
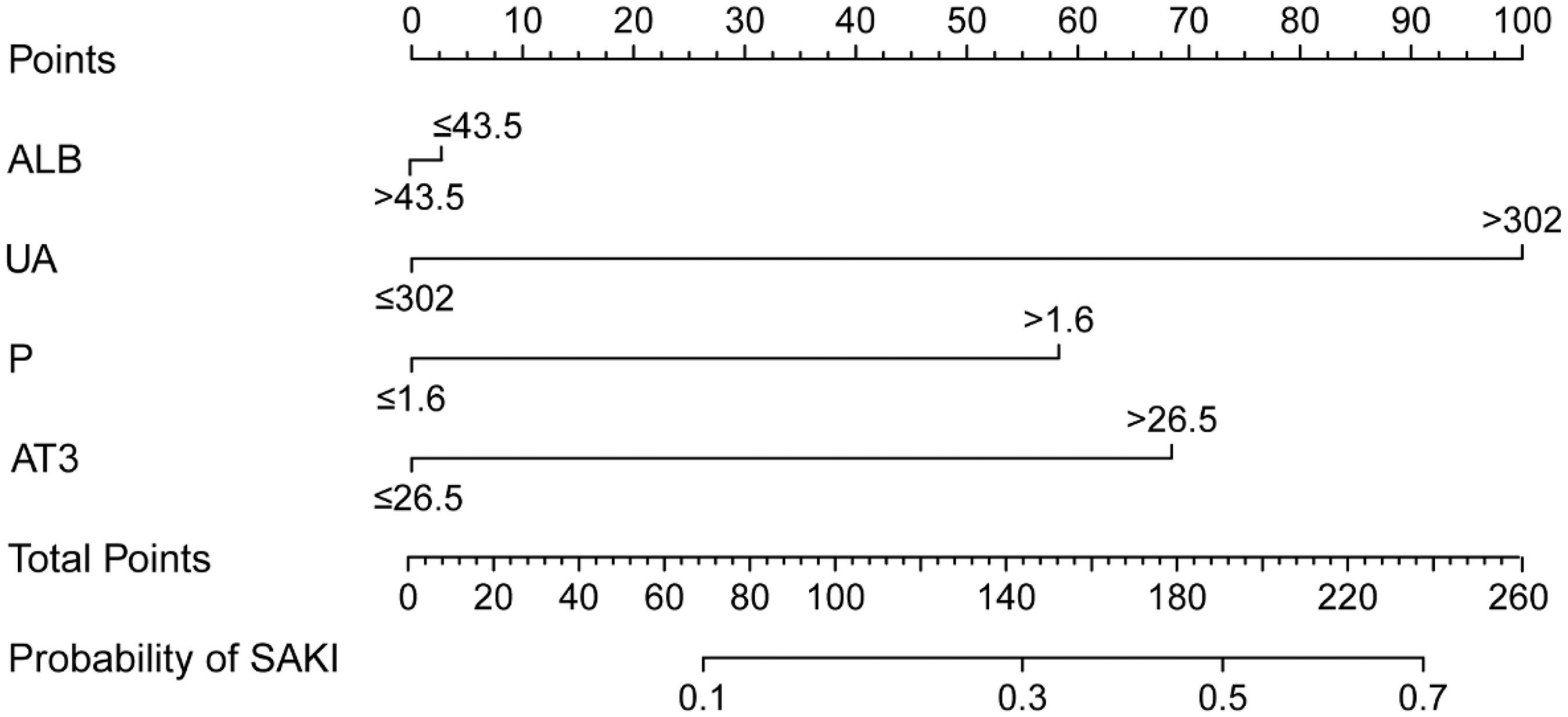
Construction of the nomogram model for predicting severe AKI in children with sepsis in the PICU. The nomogram incorporates four independent predictors: P^5+^, ALB, UA, and AT3. To use the nomogram, locate the patient's value for each variable on its corresponding axis, draw a vertical line upward to the “Points” axis to obtain the score for that variable. Sum the four scores to obtain the “Total Points,” then draw a vertical line downward from the Total Points axis to the “Probability of SAKI” axis to read the predicted probability of severe AKI. SAKI, severe acute kidney injury.

**Figure 6:**
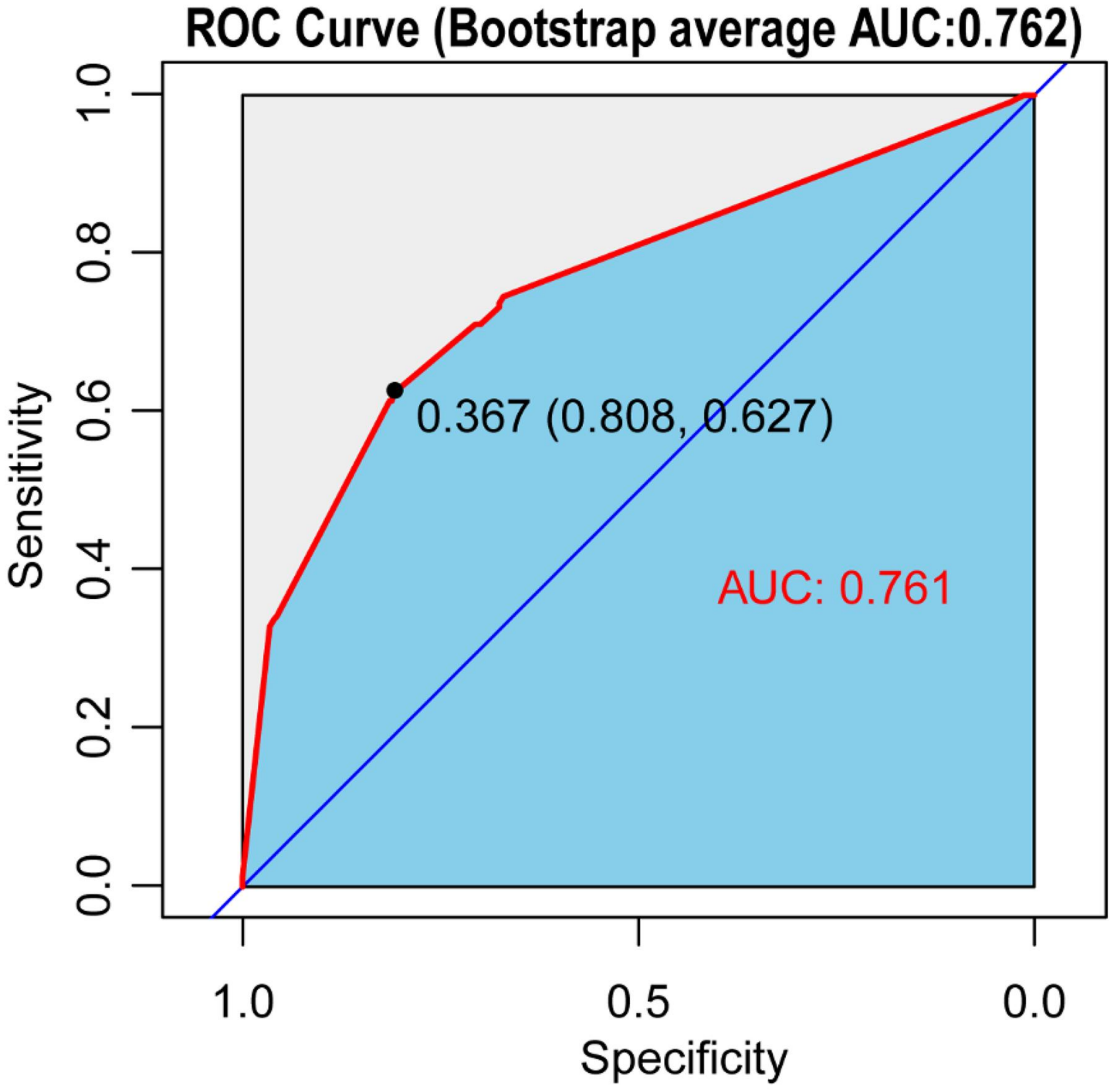
ROC curve of the prediction model for severe AKI in pediatric sepsis patients admitted to the PICU.

**Figure 7:**
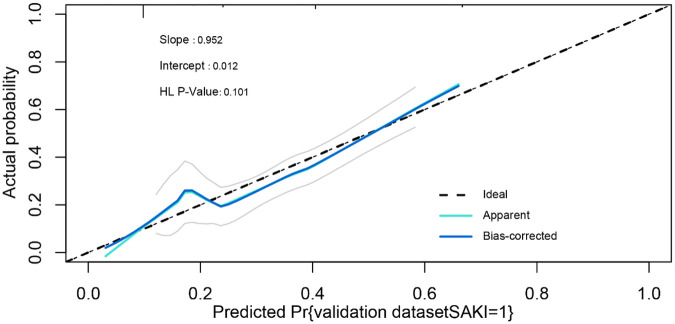
Calibration curve of the prediction model for severe AKI in pediatric sepsis patients admitted to the PICU. SAKI: Severe Acute Kidney Injury.

**Figure 8:**
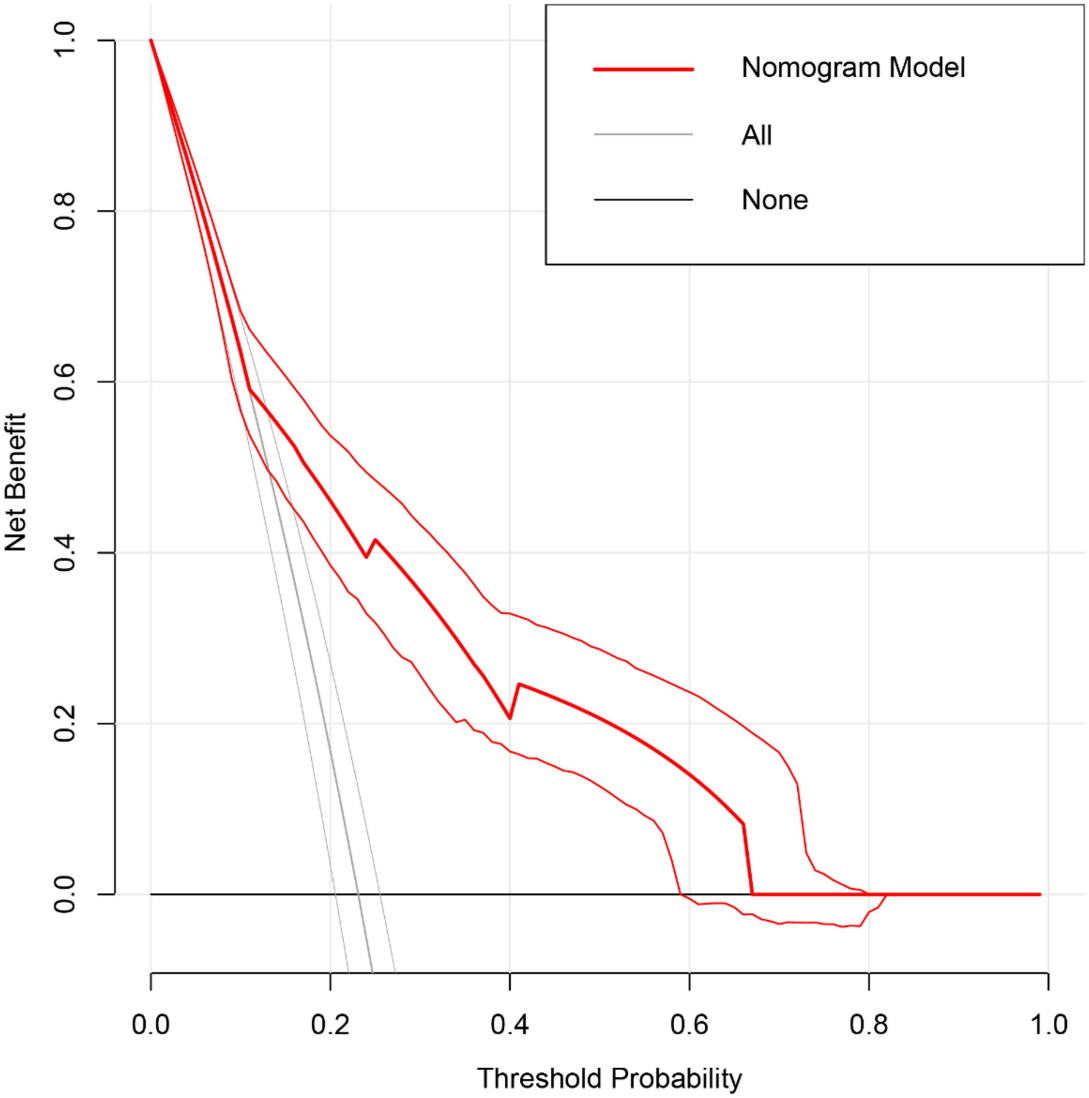
The DCA curve of the prediction model.

To enhance the usability of the model, four variables (P^5+^, ALB, UA, AT3) were identified as independent risk factors through multivariate logistic regression analysis. These variables were assigned values based on cutoff points (Table 4). A nomogram for predicting severe acute kidney injury in pediatric patients with sepsis was established using the rms package in R version 4.2.2. This transformed the regression equation into a user-friendly visual representation, improving readability and facilitating clinical use. Each variable corresponds to a score based on its value range for different patients, and by calculating the total score, the associated risk of occurrence can be obtained (Figure 5). The predictive performance of the nomogram was evaluated using receiver operating characteristic (ROC) curve analysis, which yielded an area under the curve (AUC) of 0.761 (*95%CI*: 0.724–0.797). Internal validation with 1,000 bootstrap resamples demonstrated a mean AUC of 0.762 (*95% CI*: 0.724–0.799). At the optimal cutoff value of 0.367, the model achieved a Youden's index of 0.435, with a sensitivity of 62.70%, a specificity of 80.80%, a positive predictive value of 49.50%, a negative predictive value of 87.80%, a positive likelihood ratio of 3.261, and a negative likelihood ratio of 0.462 (Figure 6). The Hosmer-Lemeshow test yielded a *P* value of 0.101, indicating no statistically significant difference between predicted probabilities and observed event rates, and thus good calibration (Figure 7). Decision curve analysis demonstrated that within the threshold probability range of 0–0.7, the nomogram provided higher net benefit than both the “treat-all” and “treat-none” strategies, suggesting its clinical utility (Figure 8).

**Table 4:** Binary cutoff values and assignments for predictive indicators.

Variable	Cutoff	Assignment
ALB	>43.5	0
	≤43.5	1
UA	≤302	0
	>302	1
P^5+^	≤1.6	0
	>1.6	1
AT3	≤26.5	0
	>26.5	1

The cutoff value was determined based on the principle of maximizing the Youden index in the univariate ROC analysis. P^5^⁺, phosphate; ALB, albumin; UA, uric acid; AT3, antithrombin III.

## 4 Discussion

Our findings demonstrate that patients with severe AKI had a mortality rate of 31.1%, 2.57 times higher than those with no clinically significant AKI, indicating that severe AKI significantly increases mortality in pediatric sepsis. This aligns with multiple retrospective studies (14, 15) and is further corroborated by a prospective study involving 128 PICUs across 26 countries, which showed that children with sepsis complicated by severe AKI had more than twice the rate of poor outcomes (death or new moderate disability) compared to those with no or mild AKI (64% vs. 30%; *P* < 0.001) (16). Consistent with this, a study focusing on elderly sepsis patients demonstrated significantly higher mortality in those with AKI compared to those without (46.15% vs. 21.95%) (17), further supporting the role of AKI as a critical prognostic determinant in sepsis. Therefore, identifying risk factors for severe AKI in pediatric sepsis may facilitate early recognition and management, potentially mitigating the development and progression of kidney injury and thereby improving clinical outcomes.

Our analysis revealed that children with sepsis complicated by severe AKI exhibited decreased levels of ALB and AT3 and elevated levels of P^5+^ and UA, with all differences statistically significant (*P* < 0.05). Multivariate logistic regression confirmed P^5+^ and UA as independent risk factors (*P* < 0.05), while ALB and AT3 served as protective factors (*P* < 0.05). These findings align well with established pathophysiological mechanisms.

Albumin, functioning as both a transport protein and antioxidant, plays multiple physiological roles, including maintaining acid-base balance, substance transport, and free radical clearance. During severe sepsis, serum albumin levels gradually decline, mainly due to capillary leakage causing albumin extravasation, preferential hepatic synthesis of acute-phase proteins at the expense of albumin, and increased catabolism under hypermetabolic conditions (18, 19). Wang et al. (20) identified low albumin as a significant risk factor for AKI in PICU sepsis patients, and a prospective study (21) demonstrated that decreasing albumin correlated with increased incidence of septic shock, AKI, acute liver injury, and 28-day mortality. From a pathophysiological perspective, albumin's protective effect largely depends on its antioxidant properties—it helps clear reactive oxygen species and binds free iron, thereby alleviating oxidative stress injury in renal tubular epithelial cells (22, 23). In addition, albumin maintains the integrity of the endothelial glycocalyx and reduces capillary leakage, indirectly preserving renal perfusion and filtration function (24, 25). Thus, low albumin is not merely a marker of sepsis severity but is directly involved in AKI progression.

Uric acid, a product of purine nucleotide metabolism, exerts an important antioxidant effect at physiological concentrations. However, when uric acid rises to supraphysiological levels, its role shifts. Jiang et al. (26) identified hyperuricemia as an independent risk factor for AKI in sepsis patients (*OR* = 4.415, *95% CI* 2.793–6.980, *P* < 0.001). Hyperuricemia contributes to kidney injury through multiple pathways: it enters renal tubular epithelial cells via urate transporters, activates NADPH oxidase, promotes excessive reactive oxygen species production, triggers the RASS system, and stimulates endothelial cells to produce inflammatory factors, leading to endothelial senescence and apoptosis (27, 28). These changes result in renal microvascular dysfunction and tubulointerstitial inflammation, directly aggravating kidney damage (29).

Phosphorus clearance depends heavily on renal function, making hyperphosphatemia a common complication of AKI that significantly correlates with increased mortality (30). Fang et al. (31) demonstrated that each 1 mg/dL increase in phosphorus was associated with 1.51–1.64 times higher AKI risk. Beyond reflecting declining renal function, hyperphosphatemia itself exerts direct nephrotoxic effects: it induces deposition of calcium-phosphate complexes in renal tubules, leads to microcalcification and tubular obstruction (32), and activates the FGF23/klotho pathway, promoting vascular calcification and further compromising renal blood flow (33). This establishes a vicious cycle in which declining kidney function reduces phosphate excretion, and hyperphosphatemia in turn exacerbates kidney injury (34, 35).

Sepsis frequently involves coagulation dysfunction (36). AT3, a small glycoprotein synthesized by the liver, inactivates thrombin and suppresses the coagulation cascade (37). During sepsis, inflammatory factors damage the vascular endothelium and activate the coagulation system, leading to excessive AT3 consumption and microvascular thrombosis. Because the kidneys are high-perfusion organs, their glomerular capillary networks are particularly prone to microthrombus formation, directly impairing renal blood flow (38, 39). Recent studies have also revealed that AT3 possesses anticoagulation-independent anti-inflammatory and cytoprotective properties, including inhibition of neutrophil recruitment and suppression of the NF-κB pathway (40, 41). Endogenous AT3 deficiency exacerbates acute kidney injury, whereas exogenous AT3 supplementation attenuates renal damage (42). Thus, a decline in AT3 drives AKI through the interconnected dimensions of coagulation, inflammation, and endothelial injury.

In our study, the prediction model incorporating P^5+^, ALB, UA, and AT3, represented by Logit(P) = −0.9121 + 1.056X_1_–0.0716X_2_ + 0.0046X_3_–0.0077X_4_, showed moderate predictive performance (sensitivity 60.10%, specificity 85.40%, PPV 55.20%, NPV 87.70%). The corresponding nomogram achieved an AUC of 0.761 (*95% CI* 0.724–0.797). We acknowledge that the relatively low sensitivity (60%) and PPV (∼55%) limit the model's suitability as a standalone clinical decision tool, as nearly 40% of severe AKI cases would be missed and half of positive predictions would be false positives. Therefore, the model should be viewed as an adjunctive early risk stratification instrument rather than a substitute for clinical judgment. Its higher NPV (87.7%) can help rule out severe AKI with reasonable confidence, thereby avoiding unnecessary monitoring; for patients with elevated predicted probabilities, closer renal surveillance is warranted, whereas immediate invasive interventions are not recommended solely based on a positive prediction.

Compared with existing tools, the PERSEVERE-II model (43) achieved an AUC of 0.95 in the derivation cohort, with 92% sensitivity and 89% specificity. The RAI (44) (≥8) identifies children at high risk for severe AKI using creatinine change and fluid overload. The pROCK criteria (45) improve specificity by approximately 20% through individualized creatinine baseline calculation. Our nomogram (AUC 0.761, sensitivity 62.70%, specificity 86.60%) has lower sensitivity but unique value as the first model integrating multi-system indicators—coagulation (AT3), nutrition (ALB), electrolyte balance (P), and renal function (UA)—into a single prediction tool. Unlike RAI and PERSEVERE-II, which rely on dynamic creatinine changes or specific inflammatory biomarkers, our model uses routine laboratory tests obtained within 24 h of admission, allowing earlier risk stratification. This feature is particularly useful in resource-limited settings. The high specificity (86.60%) and NPV (89.10%) suggest the model is better suited as a rule-out tool for identifying low-risk children. Future studies could combine our model with existing tools to leverage complementary strengths.

Several limitations should be acknowledged. First, as a single-center retrospective study focusing exclusively on PICU patients, potential selection bias may limit the model's applicability to less severe cases. Second, due to the retrospective design, we could not systematically collect data on nephrotoxic drug use and underlying diseases. Third, AKI diagnosis relied primarily on KDIGO creatinine criteria without incorporating urine output, which might have missed non-oliguric AKI or led to staging underestimation. Fourth, baseline creatinine estimation using formulas might affect staging accuracy due to individual variations (e.g., muscle mass). Fifth, although we excluded patients with pre-admission AKI, nearly half of severe AKI patients already had elevated creatinine upon PICU admission, suggesting potential delays in KDIGO criteria for early injury detection. Finally, our model requires external validation through multicenter prospective studies.

## 5 Conclusion

In summary, Severe AKI significantly increases mortality in pediatric sepsis. elevated phosphate (P^5+^), decreased albumin (ALB), elevated uric acid (UA) and reduced antithrombin III (AT3) were identified as predictive factors for severe acute kidney injury (AKI) in pediatric sepsis patients. The nomogram constructed based on these four factors demonstrated acceptable performance in both discrimination and calibration. This nomogram can visually quantify the risk of severe AKI in pediatric sepsis patients, providing a valuable predictive tool for early identification of high-risk children and supporting the development of individualized intervention strategies in clinical practice.

## Data Availability

The raw data supporting the conclusions of this article will be made available by the authors, without undue reservation.
