# Supplementary material for: Risk factors and a nomogram for predicting severe acute kidney injury in pediatric sepsis: a retrospective study
Source: Front Pediatr. 2026 Jul 20;14:1822958. doi: 10.3389/fped.2026.1822958 (PMC13429787; doi:10.3389/fped.2026.1822958)
Supplement: Supplementary file 1 [file Datasheet1.pdf]

Supplementary Materials

Table S1. Missing status of each predictor variable before interpolation (total sample size N = 987)

Variable	Number of Missing Cases (n)	Missing Proportion (%)
CRP (mg/L)	44	4.5
PCT (ng/mL)	84	8.5
Na^{+} (mmol/L)	1	0.1
Cl^{-} (mmol/L)	1	0.1
Ca^{2+} (mmol/L)	1	0.1
K^{+} (mmol/L)	1	0.1
Mg^{2+} (mmol/L)	78	7.9
P^{5+} (mmol/L)	80	8.1
TP (g/L)	1	0.1
ALB (g/L)	1	0.1
TBIL (μmol/L)	1	0.1
DBIL (μmol/L)	1	0.1
IBIL (μmol/L)	1	0.1
AST (U/L)	1	0.1
ALT (U/L)	1	0.1
TBAC (μmol/L)	1	0.1
AST/ALT	1	0.1
A/G	1	0.1
GLO (g/L)	1	0.1
BUN/CREA	28	2.8
APTT (s)	15	1.5
PT (s)	6	0.6
FIB (mg/dL)	9	0.9
TT (s)	15	1.5
INR	4	0.4
AT3 (%)	9	0.9
FDP (μg/mL)	9	0.9
D-D (μg/mL)	9	0.9

Table S2: Comparison of General Information Between Severe AKI Group and No clinically significant AKI Group

General Information	No clinically significant AKI	Severe AKI	*P* Value
Dopamine Use(n)			＜0.001
no	657(79.3%)	172(20.7%)	
yes	102(64.6%)	56(35.4%)	
Epinephrine Use(n)			0.138
no	335(79.2%)	88(20.8%)	
yes	424(75.2%)	140(24.8%)	
Mechanical Ventilation(n)			＜0.001
no	505(85.2%)	88(14.8%)	
yes	254(64.3%)	140(35.7%)	
Plasma Exchange(n)			＜0.001
no	707(80.1%)	176(19.9%)	
yes	52(50%)	52(50%)	
Hemoperfusion(n)			1
no	758(76.9%)	228(23.1%)	
yes	1(100.0%)	0(0.0%)	
Continuous Plasma Purification(n)			＜0.001
no	658(82.8%)	137(17.2%)	
yes	101(52.6%)	91(47.4%)	
Hemofiltration Use(n)			＜0.001
no	706(80.4%)	171(19.6%)	
yes	53(48.2%)	57(51.8%)	

Table S3: Comparison of Laboratory Indicators between Severe AKI Group and No clinically significant AKI Group.

Variable	No clinically significant AKI	Severe AKI	*P* Value
RBC (10^12/L)	3.62±0.83	3.68±0.87	0.38
WBC (10^9/L)	12.40±20.57	15.60±41.91	0.117
NE (10^-3)	499（230，988）	608（262.25，1163.75）	0.035
MO (10^-3)	490（210，970）	53.5（26.25，93.75）	0.267
LY (10^-3)	348±806	434±164.2	0.286
EO (10^-3)	8±21	9±25	0.367
BA (10^-3)	1（0，2）	1（0，3）	0.023

PLT (10^9/L)	259.00(129.00,399.5.50)	216.00 (98.75,332.00)	0.002
HB (g/L)	97.00(84.00,110.00)	97.00(83.00,113.00)	0.414
TBIL (μmol/L)	19.46±55.23	22.42±36.83	0.448
DBIL (μmol/L)	10.26±37.49	10.76±18.76	0.844
IBIL (μmol/L)	9.25±21.62	11.66±26.05	0.162
AST (U/L)	46.00(27.70,93.40)	72.65(30.30,262.62)	＜0.001
ALT (U/L)	28.40(18.40,60.80)	44.45(21.65,146.60)	＜0.001
TBAC (μmol/L)	7.80(4.10,16.78)	7.35(4.10,24.50)	0.694
AST/ALT	1.49(1.02,2.26)	1.71(1.14,2.62)	0.008
A/G	1.70±0.48	1.69±0.54	0.85
GLO(g/L)	21.85±6.10	21.19±6.89	0.164
